# Subxiphoid uniportal robotic thymectomy using da Vinci Xi system^†^

**DOI:** 10.1093/ejcts/ezaf127

**Published:** 2025-04-16

**Authors:** Takashi Suda, Mizuki Morota, Takahiro Negi, Daisuke Tochii, Sachiko Tochii

**Affiliations:** Department of Thoracic Surgery, Fujita Health University Okazaki Medical Center, Okazaki, Aichi, Japan; Department of Thoracic Surgery, Fujita Health University Okazaki Medical Center, Okazaki, Aichi, Japan; Department of Thoracic Surgery, Fujita Health University Okazaki Medical Center, Okazaki, Aichi, Japan; Department of Thoracic Surgery, Fujita Health University Okazaki Medical Center, Okazaki, Aichi, Japan; Department of Thoracic Surgery, Fujita Health University Okazaki Medical Center, Okazaki, Aichi, Japan

**Keywords:** robotic, thymectomy, uniportal, single-port

## Abstract

We report subxiphoid uniportal robotic thymectomy without intercostal access using the da Vinci Xi multi-port robot system. A 4-cm vertical incision was made 1 cm caudal to the xiphoid process. The AIRSEAL ROBOTIC SOLUTION, an air seal system compatible with the da Vinci port was used to insufflate CO_2_ at 8 mmHg. During port insertion, the left and right hands were crossed into the wound, with the camera, left hand, and right hand inserted in the order from the anterior chest to the dorsal side. To reduce the interference between the ports at the head, a key technique is to pull the camera port forward to prevent it from colliding with the other ports. Subxiphoid uniportal robotic thymectomy using the da Vinci Xi is a technique that combines excellent surgical visibility from the subxiphoid process, minimal invasiveness and enhanced operability provided by the robotic system

## INTRODUCTION

Endoscopic thymectomy is generally performed via the lateral intercostal space, but the usefulness of the subxiphoid approach has recently been reported [1]. The advantage of the subxiphoid approach is that the camera is inserted from the midline of the body, making it easy to obtain a view of the bilateral phrenic nerves and the upper pole of the thymus. In addition, subxiphoid uniportal thymectomy is performed with only a single incision of 4 cm or less in the subxiphoid region; hence, it does not involve the intercostal space, thus resulting in minimal pain with no chronic pain or numbness due to intercostal nerve damage [1, 2]. Conversely, in subxiphoid robotic thymectomy (SRT), a camera is inserted through a subxiphoid incision, and the arms are inserted via the lateral intercostal spaces. This method combines the excellent view from the subxiphoid with the operational ease of the robotic system, enabling complex surgeries such as vascular anastomosis [3]. Herein, we report subxiphoid uniportal robotic thymectomy (SURT) without intercostal access using the da Vinci Xi multi-port robot system (Intuitive Surgical, Sunnyvale, CA, USA).

## SURGICAL TECHNIQUE

**Figure 1:**
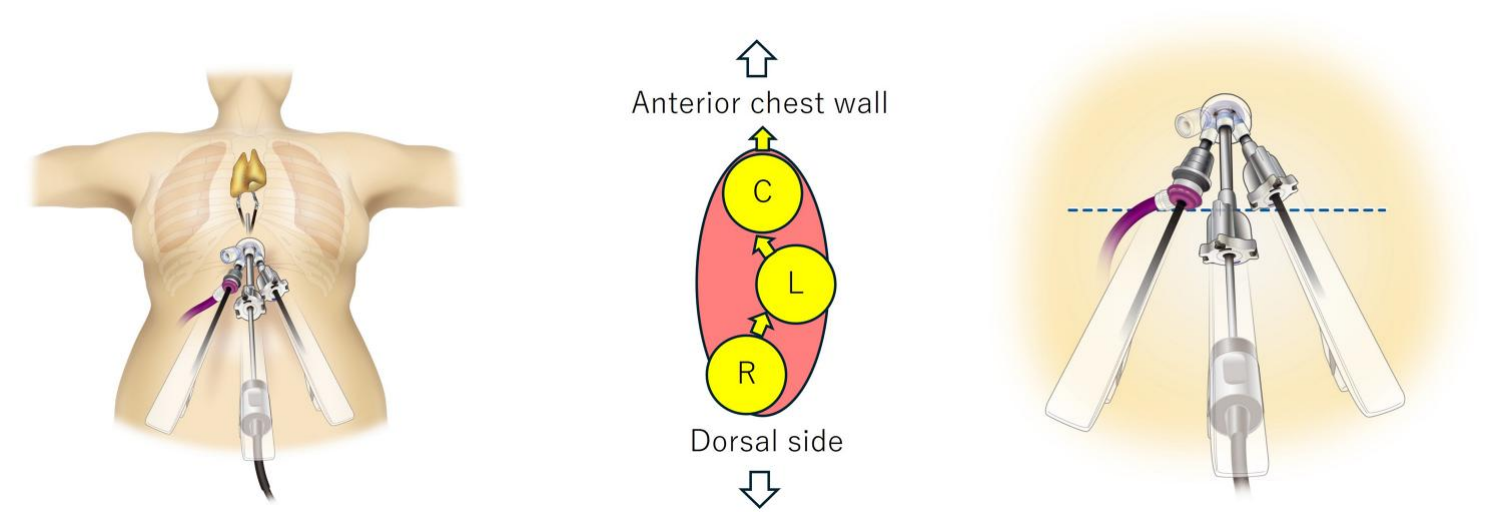
The left and right hand ports cross within the wound. The camera port is adjusted to a position where it does not interfere with the left and right hand ports.

The patient was positioned supine with arms and legs spread apart. A 4-cm vertical incision was made 1 cm caudal to the xiphoid process. A Lapsingle port (Sejong Medical Co., Paju, Korea) with a 12-mm sub-port and 3 attached 8-mm sub-ports was used. This port is transparent and allows the wound to be observed, which is useful when a da Vinci port is placed crosswise within the wound; 3 8-mm ports were used. The AIRSEAL ROBOTIC SOLUTION (ConMed, Utica, NY, USA), an air seal system compatible with the da Vinci port was used to insufflate CO_2_ at 8 mmHg. During port insertion, the left and right hands were crossed into the wound, with the camera, left hand, and right hand inserted in the order from the anterior chest to the dorsal side. To reduce the interference between the ports at the head, a key technique is to pull the camera port forward to prevent it from colliding with the other ports (Fig. 1). Additionally, to allow the left and right forceps to cross properly, it is necessary to adjust the hand control settings on the console, switching the allocation of the left and right hands. We started with a 0° camera angle and adjusted it to 30° when the camera interfered with the working field, or when the field of view was obstructed. The assistant supported the surgeon by inserting a 32 cm Schwarz curved swab (B. BRAUN, Tokyo, Japan), and a 32 cm curved suction through a small gap in the wound where the 3 ports were inserted (Video 1). The camera view from beneath the xiphoid process might pose a challenge to visualize the left pericardium; however, there was no restriction on the arm movement. Between February 2024 and December 2024, we investigated 5 consecutive cases of anterior mediastinal tumours, in which preoperative examinations confirmed that the tumours had not invaded other organs. Total thymectomy, including the removal of the upper pole of the thymus, was performed in all 5 cases. No cases required additional ports. The arm may come into contact with the abdomen; however, there is no excessive pressure, and no complications have been reported. In mediastinal surgery, we remove the drains in the operating room to allow early mobilization of the patients. However, if simultaneous lung resection is performed, we leave the drains in place due to the risk of an air leak causing bilateral pneumothorax. The drain was removed from the operating room in all cases. The median operative time was 117 (105–178) min; the median console time was 75 (33–118) min, and the median blood loss was 5 (0–5) ml. No complications or deaths occurred. Postoperative pathology revealed thymomas in 2 cases and thymic cysts in 3 cases. The Numerical Rating Scale score at rest at the first outpatient visit after discharge [median, 10 days (6–12 days)] was 0 in all patients.

This study was approved by the Fujita Health University Ethics Committee on 10 August 2021 (approval number: HM20-566). Written informed consent was obtained from all patients.

## COMMENTS

The SURT technique introduced in this report combines the minimal invasiveness of subxiphoid uniportal thymectomy with the excellent operability of SRT. Subxiphoid uniportal thymectomy has not been widely adopted by surgeons despite its remarkable benefits to the patients primarily due to the complexity of the procedure. SURT allows performing thymectomies with only a subxiphoid incision using a robotic system with excellent operability, which might enable performing even more complex surgeries. In recent years, the da Vinci SP system, designed for single-port surgery, has been introduced, with reports of its use in thymectomy [4]. However, single-port systems are expensive and have limited procedural capabilities, making them less versatile and leading to low adoption in medical facilities. In addition, obtaining a clear view of the narrow neck area with a flexible endoscope camera remains challenging. This surgical technique allows for minimally invasive subxiphoid uniportal thymectomy without the need for an expensive robotic system for single-port system, and pain is almost completely relieved within 1–2 weeks after surgery. However, challenges include the lack of a retraction arm, requiring the assistant to open the surgical field using long, curved cotton swabs or suction through the narrow wound. Moreover, the camera may sometimes interfere with the left and right hands, limiting the operation. To avoiding interference, the key is to move the left and right hands to the centre of the screen when adjusting the camera’s viewpoint. If bleeding occurs, the bleeding point is first compressed by cotton or thymus. Once temporary haemostasis is achieved, a 12 mm port with a valve is placed at the right 5th intercostal space in the anterior axillary line for improved operability and further support. The bleeding point is sutured or sealed using TachoSil^®^ (Takeda Austria GmbH, Linz, Austria). If achieving haemostasis is difficult, this approach is performed in the supine position, which allows for a prompt transition to a median sternotomy. Incision of the mediastinal pleura on both sides raises concerns about the potential for tumour dissemination, both ipsilaterally and contralaterally, as well as adhesions to the lungs. However, in 108 cases of subxiphoid thymectomy performed at our hospital over the past 10 years, ipsilateral dissemination was observed in only 2 cases, and no contralateral dissemination was recorded. Nevertheless, future evaluations of long-term results is necessary.

The key to starting this procedure is to always maintain the initial position of the port. The camera arm is adjusted to a position where the head of the camera port does not interfere with it. If the arm interferes with the procedure, ensure that the position inside the port has not been changed from the initial setting. SynchroSeal dissection forceps were used in the right hand. They are able to dissect, seal and separate and are suitable for this procedure as they require no forceps replacement. This procedure is made easier when an assistant supports the surgeon by inserting long, curved cotton swabs or forceps through the narrow port gap.

SURT using the da Vinci Xi is a technique that combines excellent surgical visibility from the subxiphoid process, minimal invasiveness and enhanced operability provided by the robotic system.

**Conflict of interest:** none declared.

## Data Availability

The data that support the findings of this study are not openly available due to reasons of sensitivity and are available from the corresponding author upon reasonable request. Data are located in controlled access data storage at Fujita Health University Okazaki Medical Center.

## References

[1] Suda T, Sugimura H, Tochii D et al Single-port thymectomy through an infrasternal approach. Ann Thorac Surg 2012;93:334–6. 22186468 10.1016/j.athoracsur.2011.08.047

[2] Suda T, Hachimaru A, Tochii D, et al Video-assisted thoracoscopic thymectomy versus subxiphoid single-port thymectomy: initial results. Eur J Cardiothorac Surg 2016;49 Suppl 1:i54-8. 26468270 10.1093/ejcts/ezv338

[3] Negi T, Morota M, Tochii D et al Initial results of uniportal and robot-assisted subxiphoid thymectomy. J Thorac Dis 2024;16:6778–88. 39552884 10.21037/jtd-24-914 PMC11565321

[4] Cheng C, Tagkalos E, Ng CB et al Single-port robotic trans-subxiphoid surgery for anterior mediastinal disease: a pilot trial. Innovations (Phila) 2024;19:268–73. 38725287 10.1177/15569845241248641

